# Supplementary material for: Ribonuclease H2 mutations induce a cGAS/STING‐dependent innate immune response
Source: EMBO J. 2016 Feb 22;35(8):831–44. doi: 10.15252/embj.201593339 (PMC4855687; doi:10.15252/embj.201593339)
Supplement: Supplementary file 1 — Appendix [file EMBJ-35-831-s001.pdf]

Ribonuclease H2 mutations induce a cGAS/STING-dependent innate immune response

Karen J. Mackenzie[1], Paula Carroll[1], Laura Lettice[1], Žygimantė Tarnauskaitė[1], Kaalak Reddy[1], Flora Dix[1], Ailsa Revuelta[1], Erika Abbondati[2] , Rachel E. Rigby[1,3], Björn Rabe[1,4], Fiona Kilanowski[1], Graeme Grimes[1], Adeline Fluteau[1], Paul S. Devenney[1], Robert E. Hill[1], Martin A. M. Reijns[1], Andrew P. Jackson[1]

1. MRC Human Genetics Unit, MRC Institute of Genetics and Molecular Medicine, The University of Edinburgh, Edinburgh, UK

2. Roslin Institute, The University of Edinburgh, Edinburgh, UK

3. MRC Human Immunology Unit, Radcliffe Department of Medicine, MRC WIMM University of Oxford, Oxford, UK

4. Institute of Biochemistry, Christian-Albrechts-University of Kiel, Kiel, Germany

Corresponding Author: andrew.jackson@igmm.ed.ac.uk

Appendix

Appendix Figure S1-4

Appendix Table S1-4

Appendix Figure S1

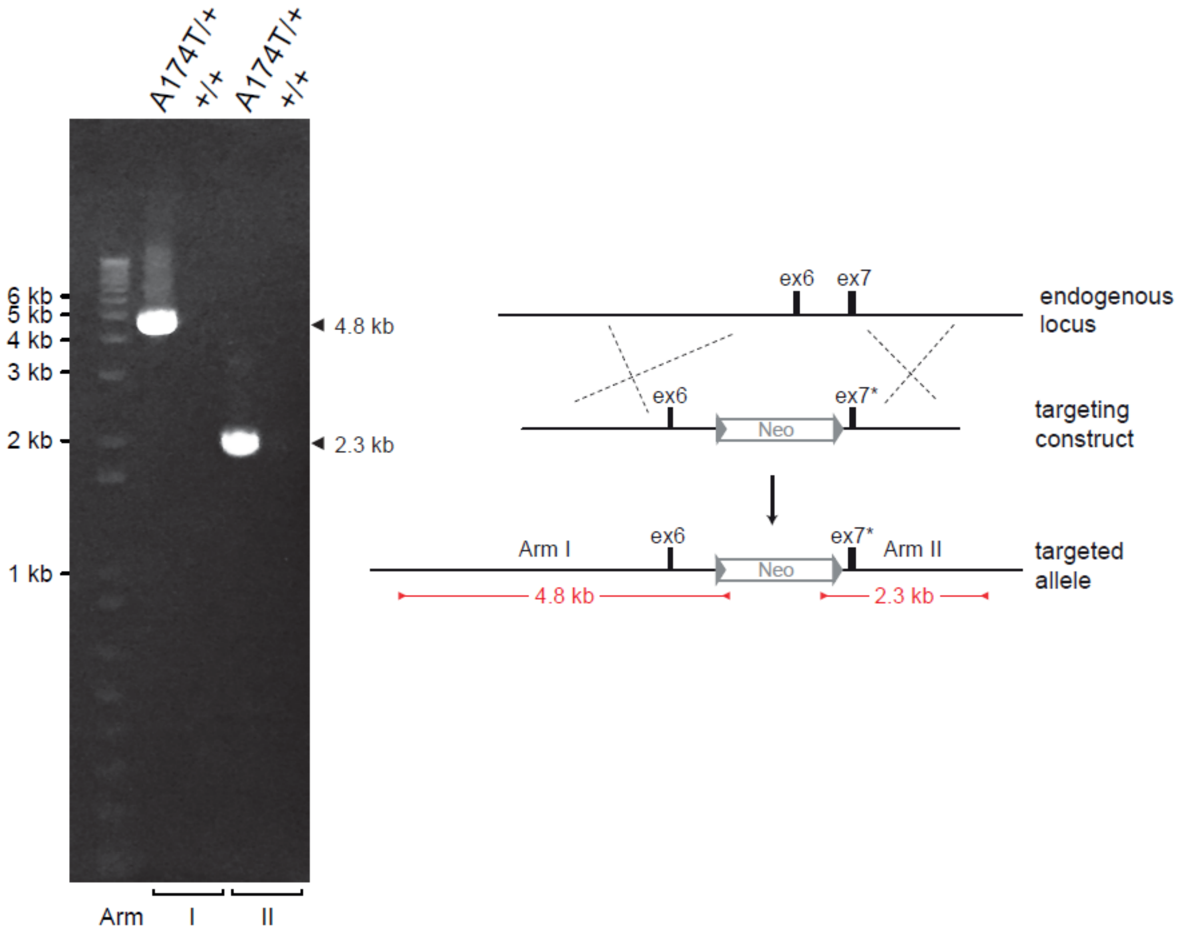

Figure S1: Long range PCR confirms correct targeting of *Rnaseh2b* exon 7 by homologous recombination

PCRs using primers (indicated by red triangles in the right schematic) amplified fragments of the expected sizes for Rnaseh2b-A174T (A174T/+), but not for wildtype (+/+) control DNA. Arm I PCR product, 4.8 kb; Arm II PCR product, 2.3 kb.

Appendix Figure S2

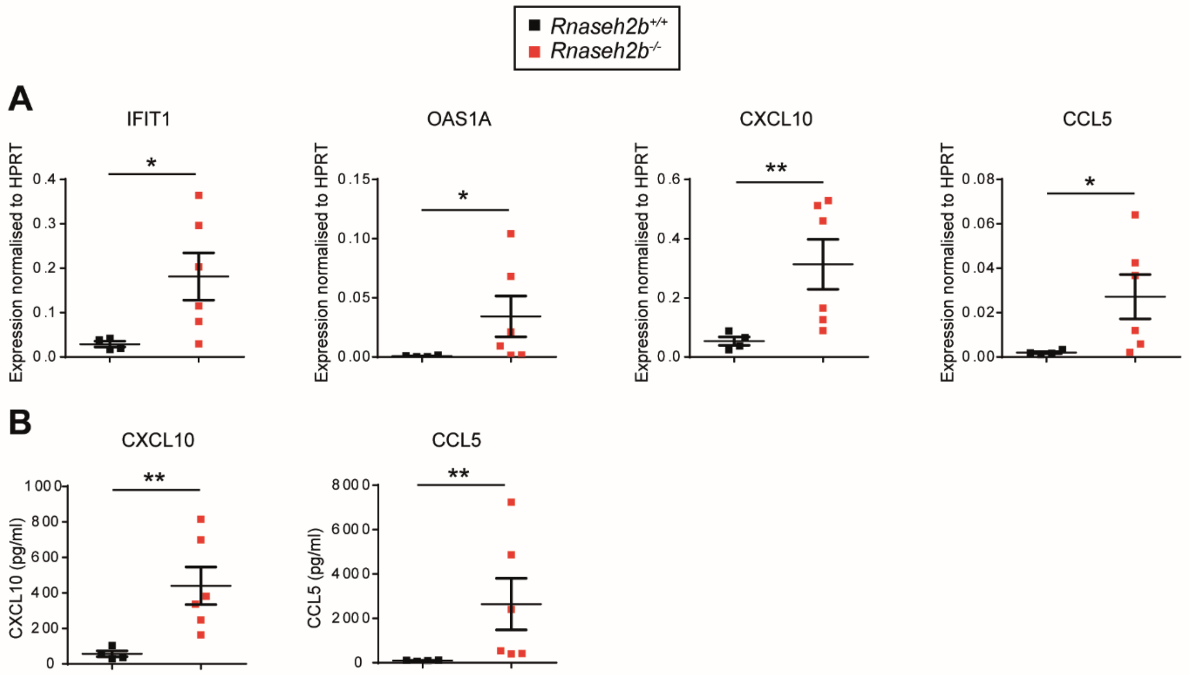

Figure S2: ISG transcript upregulation and proinflammatory cytokine secretion varies between independent *Rnaseh2b*$^{-/-}$ MEFs lines

Variation is seen in the degree of (A) ISG transcript levels and (B) secretion of proinflammatory cytokines (CXCL10 and CCL5) from independent *Rnaseh2b*$^{-/-}$ MEF lines. Each point represents the mean of three independent experiments for independent MEF lines; error bars, SEM, * = $p<0.05$, ** = $p<0.01$, Mann-Whitney U test. *Rnaseh2b*$^{-/-}$ and control MEFs on a C57/BL6 *p53*$^{-/-}$ background.

Appendix Figure S3

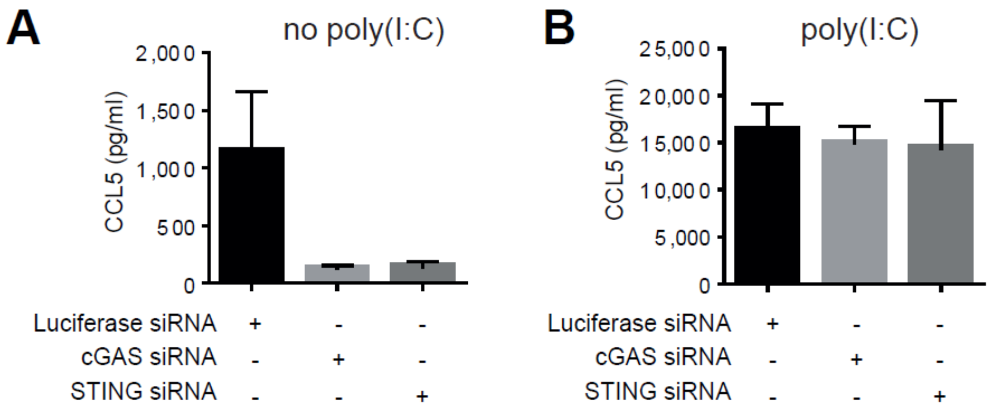

Figure S3: siRNA knockdown of STING and cGAS in Rnaseh2b$^{-/-}$ MEFs does not affect the response to poly(I:C)

The response to transfected HMW Poly(I:C) is maintained after siRNA knockdown of cGAS or STING. 48 h after luciferase, cGAS or STING siRNA treatment, *Rnaseh2b*$^{-/-}$ MEFs were further treated with (A) lipofectamine alone or (B) transfected with HMW Poly(I:C), and CCL5 assessed in culture supernatants 22 h later by ELISA. Error bars, SEM of two independent experiments.

Appendix Figure S4

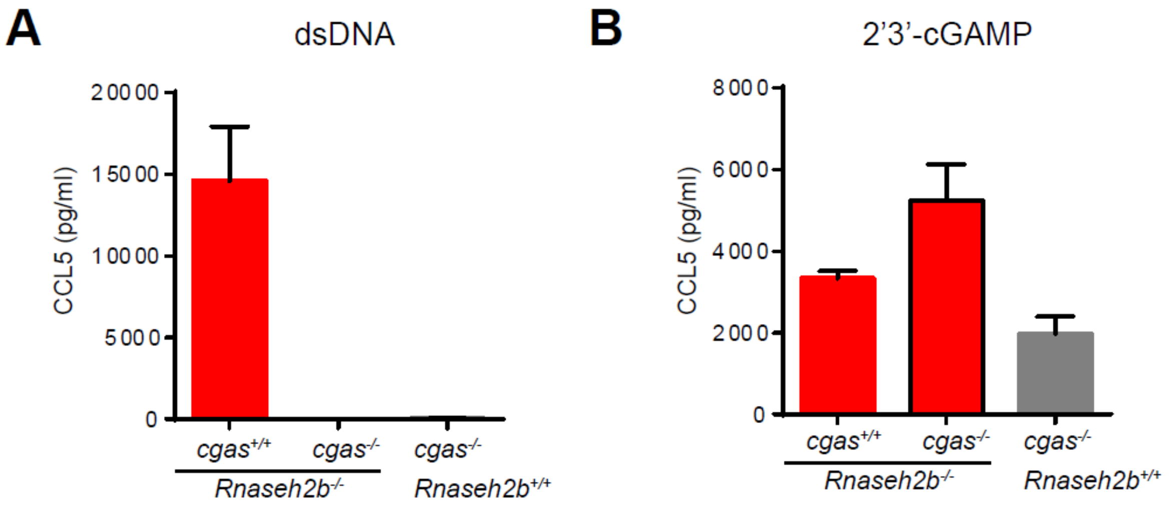

Figure S4: *cGAS$^{-/-}$ Rnaseh2b$^{-/-}$* MEFs do not produce CCL5 in response to dsDNA but remain responsive to 2’3’-cGAMP

cGAS$^{-/-}$ Rnaseh2b$^{-/-}$ MEFs (cGAS deleted by CRISPR/Cas9 in *Rnaseh2b$^{-/-}$* MEFs) do not produce CCL5 in response to dsDNA, in contrast to *cGAS$^{+/+}$ Rnaseh2b$^{-/-}$* control MEFs, but retain 2’3’-cGAMP responsiveness. *Rnaseh2b$^{-/-}$ cGAS$^{-/-}$* and *Rnaseh2b$^{-/-}$ cGAS$^{+/+}$* clones were transfected with (A) dsDNA or (B) 2’3’-cGAMP. Culture supernatant was collected 24 h after transfection and the concentration of CCL5 assessed by ELISA. An independent *cGAS$^{-/-}$* MEF line (derived from a *cGAS$^{-/-}$* embryo) was included as a control*.* Two independent experiments, n=2 *Rnaseh2b$^{-/-}$ cGAS$^{-/-}$ clones,* n=4 *Rnaseh2b$^{-/-}$ cGAS$^{+/+}$* clones, error bars, SEM of each experiment.

Appendix Tables S1-4

Appendix Table S1 shows dataset from BeadArray transcript analysis (Illumina) referred to in Fig 3C. All upregulated transcripts comparing two *Rnaseh2b*$^{-/-}$ MEF lines versus 4 *Rnaseh2b*$^{+/+}$ MEF lines are shown. ISGs as defined in corresponding references are highlighted in yellow. Appendix Table S2-3 show the primers and oligonucleotides used in this work.

Appendix Table S1. Illumina Microarray Transcriptome Analysis

SYMBOL	ENTREZGENEID	Evidence for ISG status	log(2)FC	fold change	AveExpr	t	P.Value	adj.P.Val	*Rnaseh2b* $^{-/-}$ #1	*Rnaseh2b* $^{-/-}$ #2	Control #1	Control #2	Control #3	Control #4
Ccl5	20304	Schoggins et al, 2011	3.77681029	13.70670879	7.79736004	10.7849125	4.33E-06	0.006728781	10.2567294	10.14237817	7.024565689	6.33887741	5.78120051	6.54633038
Isg15	100038882	Schoggins et al, 2011	3.44364326	10.88027609	8.26035381	9.93033309	8.11E-06	0.008015943	11.21396774	9.982666454	6.79290544	7.4762559	6.9844992	7.3650348
Ifi27l2a	76933	Schoggins et al, 2011	3.33502892	10.09122153	6.11720643	10.3210388	6.05E-06	0.007009813	8.145752018	8.632990244	5.378069126	4.80046637	5.46594371	4.57288966
Usp18	24110	Schoggins et al, 2011	2.84987731	7.209390594	6.18548829	10.9372326	3.89E-06	0.006728781	8.578706575	7.910369988	5.607778506	5.03496488	5.26477034	5.67113015
Oas1g	23960	Schoggins et al, 2011	2.54485847	5.835508878	5.2729193	14.3679447	4.70E-07	0.002084547	7.466531463	6.875939519	4.758433352	4.52461259	4.63887516	4.58358698
Myl7	17898		2.48091819	5.582526495	5.26257121	14.0020849	5.75E-07	0.002084547	7.261668079	6.81505084	4.82268576	4.48335505	4.39495773	4.52876653
Apol9a	223672	Sun et al, 2015	2.40297361	5.288921665	6.38457299	10.3125808	6.09E-06	0.007009813	8.643231501	7.712383409	5.718020969	5.80859257	5.74201012	5.83071172
Chn2	69993		2.28611858	4.877421266	5.82551691	9.38349678	1.24E-05	0.009722625	7.340319022	6.563077451	4.940267328	4.68407129	4.41795569	4.6200243
Bst2	69550	Schoggins et al, 2011	2.24727558	4.747854025	8.11661268	9.58874859	1.06E-05	0.00956606	10.01951562	9.180617919	7.181560275	7.20740045	7.48086238	7.54134166
H2-D1	14964	Saleiro et al, 2015	2.20523508	4.611496731	8.55452115	11.5027462	2.64E-06	0.0057498	9.998760218	10.11812226	8.221696368	7.83893839	7.70932455	7.64286535
Ifit3	15959	Schoggins et al, 2011	2.08573891	4.244924552	6.08444456	6.46543957	0.00018373	0.045469812	8.241978828	6.973972282	5.845544717	5.45930925	5.2451995	5.5388931
Gm8909	667977		2.04485955	4.126330995	6.69520225	10.2359055	6.45E-06	0.007009813	8.082740104	8.170426474	5.823254523	5.80989004	6.29871695	6.39503344
Lgals3bp	19039	Saleiro et al, 2015	2.03821266	4.107363584	8.73501132	8.36426731	2.92E-05	0.017616209	10.43163001	10.02620888	8.723203363	7.88919706	8.04626943	8.10415729
Oasl2	23962	Schoggins et al, 2011	2.03347596	4.09390029	6.35160204	6.79763058	0.00012958	0.038734695	8.326417946	7.202208409	5.595446825	5.94334825	5.43459934	5.94995444
Dusp4	319520		1.95966389	3.88971349	7.04380334	6.56125539	0.00016591	0.045108079	8.08167466	7.964163522	6.840282243	5.81804864	5.70540646	5.88928345
Dcn	13179		1.91513373	3.771487728	10.5404251	7.36008567	7.38E-05	0.030853791	11.22576902	11.75749582	9.930967183	9.88186966	9.17731645	9.31584146
Apol9b	71898	Sun et al, 2015	1.90879413	3.754951119	5.52378156	7.89007922	4.47E-05	0.020620537	7.396226181	6.624659854	5.306776107	4.76570422	5.24633615	5.08777909
Gdf15	23886		1.90143961	3.735857976	5.75108259	13.467195	7.79E-07	0.002119196	6.963752112	7.084805328	5.204486565	5.01012735	5.08028632	5.19645621
Chchd10	103172		1.82321233	3.53868251	8.04504032	8.08532935	3.74E-05	0.018811913	8.942755813	9.568137921	7.758067023	7.38791155	7.22180435	7.36115522
Cxcl10	15945	Schoggins et al, 2011	1.81706614	3.523639026	5.34372106	6.78620767	0.00013112	0.038734695	6.99392446	5.984103378	4.894867259	4.6441326	4.45177231	4.69701896
Hs3st1	15476		1.7033674	3.256601969	8.86806737	6.93714752	0.00011232	0.038734695	9.616742468	9.746646806	7.906486432	8.37034445	7.8223703	7.81410776
Trim30a	20128	Sistigu et al, 2014	1.64110739	3.119051525	5.19078876	6.69291747	0.00014446	0.040283256	6.849307975	5.987747501	4.850162585	4.71674606	4.56418781	4.97858496
Psmb9	16912	Schoggins et al, 2011	1.47624149	2.782229619	5.31422216	9.25806549	1.37E-05	0.009956497	6.316267493	6.385606611	5.142026292	4.6918514	4.9518005	4.71310406
Btc	12223		1.43634845	2.706350033	5.34716966	8.42371949	2.77E-05	0.017616209	6.500106299	5.968776423	4.739305721	4.87016532	4.68116466	4.90173596
Samd9l	209086	Schoggins et al, 2011	1.33387322	2.520785262	9.14455141	6.88833575	0.00011806	0.038734695	10.25210731	9.847759979	8.986765627	8.70784622	8.72552794	8.4441019
4930486L24Rik	214639		1.25743945	2.390710524	5.57544641	8.06559716	3.81E-05	0.018811913	6.46684691	6.528389831	5.267300299	5.29956095	5.38127427	5.01258015
Igtp	16145	Saleiro et al, 2015	1.25343108	2.384077409	6.91426671	6.82137625	0.00012645	0.038734695	8.112594778	7.564542696	6.518792003	6.57777454	6.52721796	6.71676614
Heph	15203		1.11434501	2.164966969	5.06080761	6.46426051	0.00018397	0.045469812	5.769112718	5.807277293	4.83688968	4.65930966	4.56944415	4.62975648
Abcc3	76408		0.92914134	1.904142354	5.02796371	7.32130413	7.66E-05	0.030853791	5.734208849	5.554228423	4.644271761	4.66235145	4.77949363	4.77419235

Refererences:

Saleiro D, Mehrotra S, Kroczynska B, Beauchamp EM, Lisowski P, Majchrzak-Kita B, Bhagat TD, Stein BL, McMahon B, Altman JK, Kosciuczuk EM, Baker DP, Jie C, Jafari N, Thompson CB, Levine RL, Fish EN, Verma AK, Platanias LC (2015) Central role of ULK1 in type I interferon signaling. Cell Rep 11: 605-617

Schoggins JW, Wilson SJ, Panis M, Murphy MY, Jones CT, Bieniasz P, Rice CM (2011) A diverse range of gene products are effectors of the type I interferon antiviral response. Nature 472: 481-485

Sistigu A, Yamazaki T, Vacchelli E, Chaba K, Enot DP, Adam J, Vitale I, Goubar A, Baracco EE, Remedios C, Fend L, Hannani D, Aymeric L, Ma Y, Niso-Santano M, Kepp O, Schultze JL, Tuting T, Belardelli F, Bracci L et al. (2014) Cancer cell-autonomous contribution of type I interferon signaling to the efficacy of chemotherapy. Nat Med 20: 1301-1309

Sun L, Miyoshi H, Origanti S, Nice TJ, Barger AC, Manieri NA, Fogel LA, French AR, Piwnica-Worms D, Piwnica-Worms H, Virgin HW, Lenschow DJ, Stappenbeck TS (2015) Type I interferons link viral infection to enhanced epithelial turnover and repair. Cell Host Microbe 17: 85-97

Appendix Table S2. Genotyping primer sequences

Genotype	Oligonucleotide sequences	Product sizes
Rnaseh2b$^{A174T/+}$	GATAGCTGACAAAGATAACTC	A174T = 460 bp
	CTGTCCATCTGCACGAGACT	WT = 221 bp
	CCTGGAAACCTGACCACC	
Rnasehb$^{tm1d/+}$	GAGCCAGCTGAGGACTCAAG	tm1d = 348 bp
	TGAACTGATGGCGAGCTCAG	WT = 307 bp
	GCTATGACAGCTTGTCTGTC	
Trp53$^{tm1Tyj/+}$	ACAGCGTGGTGGTACCTTAT	KO = 650 bp
	CTATCAGGACATAGCGTTGG	WT = 450 bp
	TATACTCAGAGCCGGCCT	
Sting/Mpys$^{+/-}$	CATTCTAATCCAGAGGTGGGAG	KO˜800 bp
	TTTCATCTGCCTTCCAGGTC	WT˜450 bp
	GCCTTCTTGACGAGTTCTTC	

Appendix Table S3. Quantitative RT-qPCR primer sequences

Name	Forward sequence	Reverse sequence
Mouse IFIT1	TCTAAACAGGGCCTTGCAG	GCAGAGCCCTTTTTGATAATGT
Mouse IFIT3	TGAACTGCTCAGCCCACA	TCCCGGTTGACCTCACTC
Mouse CXCL10	ATGACGGGCCAGTGAGAATG	ATTCCGGATTCAGACATCTCT
Mouse OAS1A	GCTGCCAGCCTTTGATGT	TGGCATAGATTCTGGGATCA
Mouse IFI44	CTGATTACAAAAGAAGACATGACAGAC	AGGCAAAACCAAAGACTCCA
Mouse CCL5	ACGTCAAGGAGTATTTCTACAC	GATGTATTCTTGAACCCACT
Mouse cGAS (Mb21d1)	GTTCAAACACAAGAAATGCACTG	GCTGACGGAGTACACAATCCT
Mouse STING (Tmem173)	TGAAAGGCTCTTCATTGTCTCTT	TGGCATCTTCTGCTTCCTAGA
Mouse HPRT	CTGGTGAAAAGGACCTCTCG	CAAGGGCATATCCAACAACA

Appendix Table S4. siRNA oligonucleotide sequences

Name	Target sequence
Mouse sicGAS (Mb21d1)	GGAUUGAGCUACAAGAAUA<br>AGAAAUCUCUGUGGAUAUA<br>GAAGAUCCGCGUAGAAGGA<br>GCUAAGAAGCCGUCCGCGA
Mouse siSTING (Tmem173)	GCAUCAAGAAUCGGGUUUA<br>AACAUUCGAUUCCGAGAUA<br>CAAAUCACACUCUGAAGUA<br>CGAAAUAACUGCCGCCUCA
siLUC	CUUACGCUGAGUACUUCGA
