# Supplementary figures and images for: Ribonuclease H2 mutations induce a cGAS/STING‐dependent innate immune response
Source: EMBO J. 2016 Feb 22;35(8):831–44. doi: 10.15252/embj.201593339 (PMC4855687; doi:10.15252/embj.201593339)

Source Data Figure 1

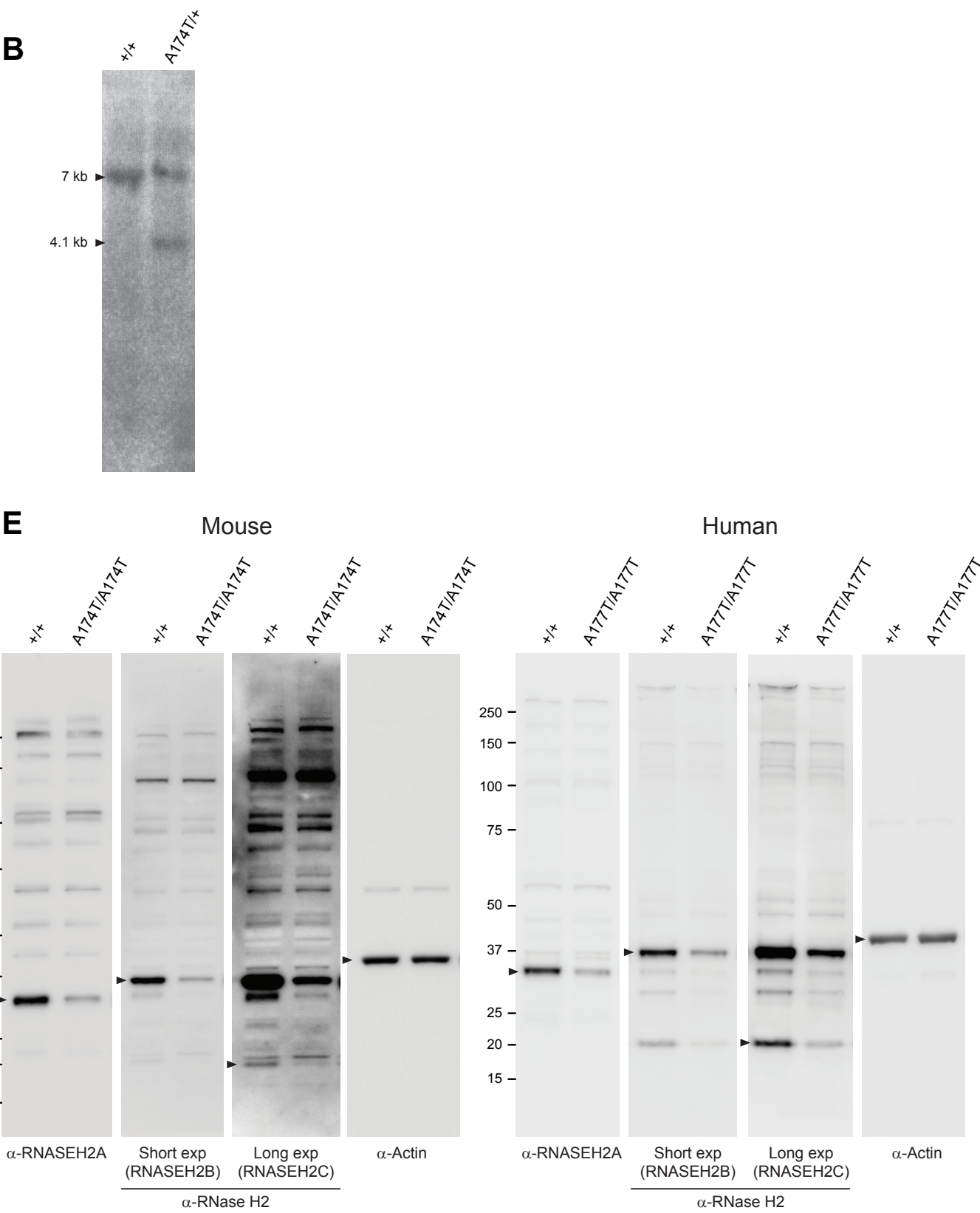

Supplement: Supplementary file 3 — Source Data for Figure 1 [file EMBJ-35-831-s002.pdf]
